# Financial Health of People Living With Dementia and Their Informal Care Partners: Protocol for a Mixed Methods Study

**DOI:** 10.2196/47255

**Published:** 2023-07-11

**Authors:** Eli Robert Boone, Heiley Tai, Ali Raich, Amulya Vatsavai, Annie Qin, Kayla Thompson, Mohini Johri, Ruitian Hu, Vishnukamal Golla, Melissa Harris-Gersten

**Affiliations:** 1 Duke-Margolis Center for Health Policy Durham, NC United States; 2 Sanford School of Public Policy Duke University Durham, NC United States; 3 Trinity College of Arts & Sciences Duke University Durham, NC United States; 4 Duke University School of Medicine Durham, NC United States; 5 Fuqua School of Business Duke University Durham, NC United States; 6 Duke Clinical and Translational Science Institute Duke University Durham, NC United States; 7 Durham Veterans Affairs Health Care System Durham, NC United States; 8 Department of Surgery Duke University Durham, NC United States; 9 School of Nursing Duke University Durham, NC United States

**Keywords:** avoiding care due to cost, care rationing, cost, delayed care due to cost, dementia, economic strain, financial burden, financial distress, financial hardship, financial strain, financial stress, financial toxicity, MCI, mild cognitive impairment, out-of-pocket costs, out-of-pocket spending

## Abstract

**Background:**

There is a growing body of academic literature focusing on the significant financial burdens placed on people living with cancer, but little evidence exists on the impact of rising costs of care in other vulnerable populations. This financial strain, also known as financial toxicity, can impact behavioral, psychosocial, and material domains of life for people diagnosed with chronic conditions and their care partners. New evidence suggests that populations experiencing health disparities, including those with dementia, face limited access to health care, employment discrimination, income inequality, higher burdens of disease, and exacerbating financial toxicity.

**Objective:**

The three study aims are to (1) adapt a survey to capture financial toxicity in people living with dementia and their care partners; (2) characterize the degree and magnitude of different components of financial toxicity in this population; and (3) empower the voice of this population through imagery and critical reflection on their perceptions and experiences relating to financial toxicity.

**Methods:**

This study uses a mixed methods approach to comprehensively characterize financial toxicity among people living with dementia and their care partners. To address aim 1, we will adapt elements from previously validated and reliable instruments, including the Comprehensive Score for Financial Toxicity and Patient-Reported Outcomes Measurement Information System, to develop a financial toxicity survey specific to dyads of people living with dementia and their care partners. A total of 100 dyads will complete the survey, and data will be analyzed using descriptive statistics and regression models to address aim 2. Aim 3 will be addressed using the process of “photovoice,” which is a qualitative, participatory research method that combines photography, verbal narratives, and critical reflection by groups of individuals to capture aspects of their environment and experiences with a certain topic. Quantitative results and qualitative findings will be integrated using a validated, joint display table mixed methods approach called the pillar integration process.

**Results:**

This study is ongoing, with quantitative findings and qualitative results anticipated by December 2023. Integrated findings will enhance the understanding of financial toxicity in individuals living with dementia and their care partners by providing a comprehensive baseline assessment.

**Conclusions:**

As one of the first studies on financial toxicity related to dementia care, findings from our mixed methods approach will support the development of new strategies for improving the costs of care. While this work focuses on those living with dementia, this protocol could be replicated for people living with other diseases and serve as a blueprint for future research efforts in this space.

**International Registered Report Identifier (IRRID):**

DERR1-10.2196/47255

## Introduction

There are significant financial burdens placed on people living with chronic, complex medical conditions and their care partners. The growing body of evidence primarily focuses on people with cancer and the subsequent adverse effects of financial hardship due to the unchecked rising costs of cancer care [1,2]. This treatment-related financial strain of cancer care for people diagnosed and their family members has been coined financial toxicity. Financial toxicity can impact multiple domains of life, including behavioral (eg, rationing medications or medical care due to costs), psychosocial (eg, depression associated with medical costs), and material (eg, bankruptcy, loss of income, and debt) [3-5]. It can take the form of both direct costs (eg, copays for appointments and medication) and indirect costs (eg, lost income from a patient or care partner’s inability to work).

There is mounting evidence that people living with dementia and their care partners face limited access to health care, employment discrimination, income inequality, and higher burdens of disease [6]; however, the majority of evidence surrounding financial toxicity has focused on the population with cancer [7]. Previous research highlights the high cumulative financial burden of dementia (particularly in the final years of life) [8-10]. Other studies have quantitatively examined the relationship between financial difficulty and care partner mental health [11]. Yet few studies have combined qualitative and quantitative methods to examine the complex behavioral, psychosocial, and material impacts of dementia and dementia-related care costs. There remains limited knowledge surrounding how these domains of financial toxicity are experienced by people living with dementia and their families, this knowledge is essential to inform system-level solutions. The Comprehensive Score for Financial Toxicity (COST) measure is a reliable screening tool within the cancer space, but a similar metric does not exist within the dementia space [12].

The purpose of this study is to comprehensively characterize the financial burdens experienced by people living with dementia and their care partners using a mixed methods approach. This study is guided by the following aims: (1) adapt a survey to capture financial toxicity in dyads of people living with dementia and their care partners, (2) characterize the degree and magnitude of different components of financial toxicity among people living with dementia and their care partners (eg, out of pocket expenses, financial stress, and retirement savings), and (3) empower the voice of people living with dementia and their care partners through images and critical reflections of their perceptions and experiences related to financial well-being and hardship due to dementia using “photovoice,” a creative, qualitative method. Results of this study will support the identification of primary drivers of financial toxicity for potential system-level solutions to mitigate patient burden.

## Methods

### Study Design

**Figure 1:**
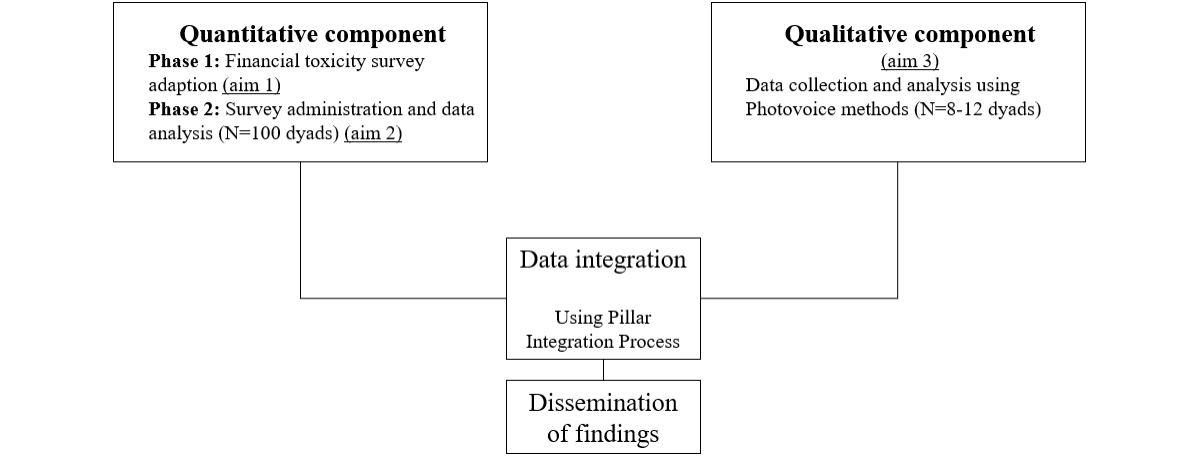
Quantitative and qualitative components and data integration overview.

This study will use a convergent mixed methods design to comprehensively characterize financial toxicity among people living with dementia and their care partners. Quantitative methods include survey adaptation (aim 1) and administration and analysis (aim 2). Qualitative photovoice methods will be used to address aim 3. Quantitative and qualitative data will be collected concurrently within a similar time frame, analyzed independently, and then integrated using a validated, joint display table approach called the pillar integration process (PIP) [13]. (See Figure 1 for an overview visualization of quantitative and qualitive component data integration)

### Quantitative Methods

A financial toxicity survey will be adapted to capture sociodemographic data, out-of-pocket expenses, and cost-coping strategies of the people living with dementia and their primary care partners. Adapted elements from validated instruments include the Patient-Reported Outcomes Measurement Information System (PROMIS) [14], COST [12], the Institute for Medical Technology Assessment Productivity Cost Questionnaire (iPCQ) [15], and the Cost of Care Index [16]. (See Table 1 for adapted elements from validated instruments included in the survey)

The survey will then be administered to 100 participant dyads of people living with dementia and their care partners. The study includes a projected sample size of 100 dyads (200 total participants). We chose a sample size of 200 for our survey study based on a power analysis conducted to detect even a small effect size (Cohen *d*=0.2) with low to moderate variability of SD 0.5-1.0. The power analysis indicated that a sample size of 200 would be sufficient to detect this small effect size with 80% power at a significance level of .05 (See Table 2 for power analysis and sample size calculations).

**Table 1:** Concepts, measurement instruments, psychometric properties, and references.

Concept and measurement instruments	Psychometric properties	References
General health of person living with dementia and care partner: Perceived General Health (Likert scale; 1 item) Mental health of person living with dementia and care partner: Perceived Mental Health (Likert scale; 1 item)	Internal consistency: Cronbach α of .73-.81 have been reported for physical and mental health reporting items [17] Test-retest reliability: ICCs^a^ were greater than 0.85 for physical and mental health scores [18]. Construct validity: physical function measures have high correlations with conceptually related measures ranging from 0.43 to 0.82, demonstrating strong convergent validity [19]. Discriminant validity: Patient-Reported Outcomes Measurement Information System physical function measures show weak correlations with dissimilar domains, indicating good discriminant validity. [20]	Patient-Reported Outcomes Measurement Information System Questionnaire [14]
Financial toxicity for dyad: Perceived Likelihood of Various Financial Costs (Likert scale; 5 items)	Internal consistency: Cronbach α of .73-.86 have been reported for general financial situation and impact of illness on financial situation items [21] Test-retest reliability: cost measures have been reported to have ICC of 0.80 [12] Construct validity: the Comprehensive Score for Financial Toxicity measures of financial toxicity are shown to have good predictive validity and be multidimensional in a sample of individuals with chronic conditions [22] Discriminant validity: discriminant validity has been reported as acceptable when compared to the Financial Index of Toxicity and Breast Cancer Finances Survey Inventory [23]	Comprehensive Score for Financial Toxicity tool [12]
Amount of missed work for the person living with dementia and for the care partner: Perceived Missed Work (Likert scale; 1 item)	Internal consistency: interitem correlation has been reported to range from 0.42 to 0.62 for productivity cost items, including absenteeism [24]. Test-retest reliability: ICCs for absenteeism have been reported as greater than 0.88, indicating overall good reliability [24]. Construct validity: confirmatory factor analysis found a 3-component solution accounting for 82% of the total variance, similar to the original study of the Institute for Medical Technology Assessment Productivity Cost Questionnaire [24] Discriminant validity: discriminant validity has been reported as acceptable when included in a comprehensive questionnaire [25]	Institute for Medical Technology Assessment Productivity Cost Questionnaire [15]
Financial toxicity of dementia care for dyad: Perceived Likelihood of Various Financial Costs (Likert scale; 5 items)	As the Cost of Care Index is not a specific instrument, it does not have standardized psychometric properties such as reliability or validity. It is primarily used to compare the relative cost of living and health care expenses in different geographies.	Cost of Care Index [16]

^a^ICC: intraclass correlation coefficient.

**Table 2:** Power analysis and sample size calculations.

	Low variability (SD 0.5)	Moderate variability (SD 1.0)	High variability (SD 1.5)
Small (Cohen *d*=0.2)	125	252	405
Medium (Cohen *d*=0.5)	32	64	102
Large (Cohen *d*=0.8)	14	28	43

Eligible participants with dementia will (1) be at least 40 years old and (2) have a diagnosis of dementia of any type (determined by self- or proxy-report by caregiver). The age range of 40 years and older was chosen, as it provides a more inclusive view of the population with dementia and has been shown to be a critical, but less understood time period in dementia-related pathology [26,27]. Eligible care partners must (1) be at least 21 years old; (2) identify as the primary care partner of the person with dementia; and (3) be able to read and speak English. Dyads must live together in the same household and will be excluded if they reside in an assisted living home, nursing home, or other long-term care setting. The survey can be completed by the dyad together or by the caregiver on behalf of the family unit.

Dyads will be recruited through several local, regional, and national dementia- and caregiver-specific community support organizations, including the Duke Dementia Family Support Program, Dementia Inclusive Durham, Durham Center for Senior Life, Family Caregiver Alliance, and the National Council of Dementia Minds. Study information and flyers will be shared through web-based and in-person events offered by these organizations. Study information will also be shared through a national social media campaign using Facebook and Reddit. Interested individuals will be able to access the eligibility screening electronically using a link or QR code or contact the study team directly to determine eligibility. Our social marketing plan was developed with guidance and support from the Duke Clinical and Translational Science Institute Recruitment Innovations Center. We will use an adaptive recruitment method and review recruitment and enrollment every week through weekly team meetings. Changes will be made to recruitment strategies as necessary to reach our projected samples.

If eligible, participants will complete the survey electronically through REDCap’s survey distribution platform or by hardcopy depending on their preference. Participant responses will auto-populate to the study’s REDCap database. All participants will review a study informed consent form embedded in the electronic survey, or as a hardcopy. Completion of the survey will be considered implied consent to participate in the study.

### Data Analysis

The data analysis for this survey will involve both descriptive and inferential statistical analysis. Descriptive statistics will be used to summarize the background demographics and qualitative responses about financial hardship. Inferential statistics, such as the Wilcoxon rank-sum test, logistic regression, and correlation analyses will be used to examine the relationships between financial health, demographic variables, and additional survey elements. Results will be reported with appropriate measures of central tendency and dispersion, and statistical significance will be determined at an α level of .05. Stata (StataCorp) will be used to manage, analyze, and create graphical presentations of data.

### Qualitative Methods

Photovoice is a highly creative, participatory research method that involves photography, reflection, and in-depth discussion to capture the lived experience and empower the voice of participants [28]. Photovoice methods were selected for this study as reflection on photos and photo-elicitation is an important approach to more actively engage people living with dementia by allowing them more opportunities to share their lived experiences using verbal communication, as well as visual cues [29,30].

The qualitative component of this study will engage 8-12 dyads of people living with dementia and their care partners recruited through the same channels as the quantitative component. The same eligibility criteria will be used for the qualitative component as is used for the quantitative survey with two additions: (1) participants with dementia in the qualitative component must also be able to express themselves verbally in English and (2) dyads must have access and ability to use a phone or internet. Dyads who complete the quantitative survey can also participate in the qualitative component, provided they meet the eligibility criteria. A study informed consent form will be sent to all participants and verbal consent will be obtained by telephone from all dyads before data collection.

All consenting dyads will attend a series of 6-8 group discussion sessions using the photovoice participatory research approach. The photovoice methodology includes taking photos, discussing these photos with a group, selecting photos to caption, and grouping photos and captions thematically (Table 3) [31,32]. Photovoice was chosen as the preferred qualitative research method because people living with dementia may be limited in their ability to engage verbally, and photos can allow them to communicate aspects about their self and daily living [33]. Given that financial changes can be mundane and ingrained into everyday life, photos may be well-positioned to capture the topic of financial toxicity. Furthermore, photo elicitation also has been shown to enable caregivers of individuals living with dementia to share a more nuanced look into their own experience [34]. Participants will be encouraged to use their own cell phones or digital cameras to take photos. Disposable cameras will be provided to participants without phones or cameras. No photos will be kept by the study team without written permission from participants. Ownership and copyright of the photos will remain with the participants, and media release forms will be obtained as needed for the dissemination of findings. Qualitative analysis occurs through interactive and iterative group discussions throughout the photovoice process. Data analysis will occur in the following ways:

- Coding selected photographs and calculating a summary frequency count by theme.
- Visual analysis of selected photographs, involving (1) descriptions of photo production techniques, for example, spontaneous or planned photography, existing photos, and photos taken by someone other than the participant; (2) thematic analysis of how the subject matter, caption content, or photo group themes relate to the broader topic [35]; and (3) structural narrative analysis of the photo and caption together to understand how participants make sense of their realities and experiences through photography [35].

**Table 3:** Overview of photovoice sessions and procedures. Procedures achieved through each session may vary depending on the group’s process. This table is meant to provide an overview and preliminary plan but may be adapted throughout the study.

Photovoice group sessions	Description
1	The research team will introduce participants to the overall photovoice process and the group will broadly discuss the meaning of financial health and well-being.
2	The research team will introduce the ethics of photo taking for research purposes and basic photography skills. As a group, participants and the research team will brainstorm ideas for photos that participants could take in their daily lives to capture financial well-being and hardship.
Between sessions 2 and 3	People living with dementia and care partners will take photographs between sessions to document their own experiences with financial well-being and financial hardship. From the photos taken, dyads will select 3-5 for group discussion.
3 and 4	People living with dementia and care partners will be asked to share and discuss their photos. They will be asked to describe how each photo relates to financial well-being and hardship, what it was like to take each photo, and what it feels like to reflect on the photo through group discussion. Sharing and discussion of photos will occur 1-on-1 with a study team member, in small groups, or with the larger group. A trained research team member will take detailed notes on these discussions. Post-group summaries will be prepared after each group by a trained research team member.
5	Participants will work with the research team to write or dictate narrative captions and titles for each photo they shared.
6 and 7	As a group, participants and research team members will group photos into themes that cluster together conceptually. The team will analyze photos, titles, captions, previous session notes, and postgroup summaries to identify emergent themes. Consensus on final themes and theme titles will be achieved through in-depth discussion.
7 and 8	Participants and research team members will work together to prepare materials to present photovoice findings through a public exhibit of photographs and captions. This exhibit will be designed to engage broad audiences to encourage dialogue among people living with dementia and their families, researchers, community leaders, health care professionals, policymakers, decision-makers, and others.

### Data Integration Process

Quantitative and qualitative data will be analyzed independently and then integrated using a validated mixed method, joint display table approach called PIP. The PIP includes 4 stages to integrate mixed methods data after it has been analyzed independently (See Table 4, Table 5, and Textbox 1 for stage descriptions, exemplar, and emergent pillar themes) [13]. The PIP is designed to not only verify or illustrate discrepancies between quantitative results and qualitative findings, but also expand findings through identification of emergent pillar themes. Pillar themes emerge by rigorously comparing and contrasting quantitative results and qualitative findings.

**Table 4:** Pillar integration process stages.

Pillar integration process stages	Description
Stage 1: Listing	Raw data and coded or grouped data are listed in a joint display table (Table 5) in the quantitative data and categories columns, or the qualitative codes and qualitative categories columns.
Stage 2: Matching	Quant data and qualitative codes that reflect similar content, patterns, or qualities are horizontally aligned to match quantitative results to qualitative findings.
Stage 3: Checking	Data cross-checked for completeness and appropriate matching.
Stage 4: Pillar building	Findings in the quantitative and qualitative columns are compared and contrasted, and gaps are identified to analyze patterns, insights, and emergent findings to build inferences from the findings as a whole, then added to the pillar building themes column.

**Table 5:** Pillar integration process exemplar table. Concept: health-related financial hardship among people living with dementia and their care partners; goal: illustrate points of convergence in quantitative results with qualitative findings, identify discrepancies and gaps, and expand findings to elucidate potential strategies to reduce financial hardship among dyads living with dementia

Quantitative data	Quantitative categories	Pillar building themes	Qualitative categories	Qualitative codes
Raw data (percentages, frequencies, means, cross-tabulations)	Coded or grouped data abstracted from raw data (categories abstracted from raw data)	Integrated themes identified through the pillar building process	Coded or grouped data (themes and categories)	Raw data (narratives, captions, individual photographs, notes)

**Pillar integration process data and emergent pillar themes.**

**Hypothesized example of PIP data and emergent pillar theme**

- 30% of lower-income dyads report that they do not have enough money in savings, retirement, or assets to cover the costs of the partner living with dementia care, compared to 10% of higher-income dyads.
- Higher use of savings to pay for care for lower-income dyads.
- Targeted strategies are needed that include alternatives to using savings to pay for dementia related-care for families in lower income brackets.
- Unanticipated use of savings.
- Caption of a photo taken by photovoice participant with dementia of a hospital bill: “Not my plan for retirement savings.”

### Dissemination of Findings

Findings will be disseminated through scholarly outlets (eg, abstracts, manuscripts, symposiums, and presentations), as well as to the broader public through news and social media. A web-based or in-person photovoice exhibit will be held to raise awareness of financial toxicity in this population. The exhibit’s intent will be to inspire discussion and actionable solutions to improve the lived experiences of people living with dementia and their care partners. Further brainstorming sessions may be planned, depending on the interest of attendees.

### Ethics Approval

This study protocol was reviewed and approved by the Duke Health Institutional Review Board (PRO00111170). 

### Consenting Procedures

#### Quantitative Component

In place of written or verbal consent, participants will be asked to review a study information sheet embedded at the beginning of the survey. The study information sheet indicates participation is voluntary and that participants can stop the survey for any reason at any time. Completion of the survey implies voluntary consent. The study information sheet will include contact information that participants can use to contact the study team with any additional questions. 

#### Qualitative Component

All participants will provide their consent verbally to participate in the photovoice project. Potential participants will review a study consent form with a research team member by phone who will assess participants’ understanding of participation and answer any questions that participants have regarding the study. After reviewing the consent form, participants will be asked verbally if they wish to participate in the study. Both members of the dyad must provide verbal consent. Date of verbal consent and staff member who received consent will be documented. The research team member obtaining consent will let participants know that they can ask questions regarding consent or withdraw participation at any time throughout the study.

#### Understanding of Participation

To demonstrate a sufficient understanding of study participation, a trained research team member designated to obtain consent will use the teach-back method [36], where they will ask potential participants to recall or explain topics that were discussed in the study information sheet, using their own words. These questions are meant to prompt a discussion [36]. The designated research team member will ask the following questions to assess comprehension of informed consent: (1) “This is a research study, do you have to participate?” (2) “What would you do if you change your mind about participating in the study?” (3) “There are some risks involved in this study. Can you tell me about the risks discussed?” (If they do not remember, they are prompted to review them again), and (4) “Do you still want to participate after hearing all the details?” If the person living with dementia or the care partner participant cannot demonstrate comprehension of study participation or necessary cognitive ability for study participation or both as assessed by the research team member, the dyad will not be included in the study.

### Confidentiality

To protect participants’ confidentiality, we will limit the amount of protected health information and identifiable information that is collected. Data management will strictly adhere to the Duke University Health System Institutional Review Board guidelines and federal privacy regulations. All participant data (eg, questionnaires, photos, captions, discussion notes, themes, and categories) will be deidentified, where each participant will receive a 3-digit identification number that will be used to label their data materials. Only the study principal investigators will have access to the key linking identification numbers to participants’ names. No identifiers will be used in reports, materials, or presentations that originate from this study. For publication purposes, only group data will be published. All data will be stored in a secure Duke University manner (secure drives, identifiers separate). More specifically, all recorded information will be documented in an electronic format. All electronic data files will be stored only on a password-protected secure file server (located behind a firewall) using a Duke University secure file server, or on a secure REDCap study database. Nonelectronic files will be immediately entered into the study database upon receipt, then shredded.

### Compensation

Dyads that complete the survey will be able to follow a separate link at the end of the survey to provide their contact information to receive a US $25 gift card as compensation. These responses will not be linked to the finance survey responses. Participant dyads in the photovoice component will receive a US $100 check for compensation.

## Results

### Quantitative Component

This study is ongoing with survey data collection anticipated to be completed by December 2023. Quantitative results from survey analysis are expected to describe the extent to which dyads experience financial toxicity and correlation across sociodemographic identities, out-of-pocket expenses, and cost-coping strategies.

### Qualitative Component

The study team anticipates a comprehensive collection of photographs and narrative captions from each photovoice session, compiled into a poster for exhibition. Anticipated results from the data integration process are emergent themes pertaining to potential strategies to reduce financial hardship among dyads living with dementia (see Table 5) [13]. Emergent themes will be highlighted in the photovoice exhibit and used to spark discussion among the research team and attendees.

## Discussion

### Overview

Financial toxicity has garnered more of the limelight in recent years; however, the academic and policy focus has centered on people diagnosed with cancer. While rising financial costs and associated financial burden on health systems are frequently studied, there remains a dearth of evidence on the impact these costs might have on the individual level. Longitudinal and chronic diseases have rarely found a place in the academic literature, especially with regard to the financial impact on the patient and their family. Dementia is one particularly important chronic disease process with an estimated prevalence of 7 million people living with dementia in the United States, and the number is projected to rise to 12 million by 2040 [37]. Our study would be one of the first to use a mixed methods approach to characterize financial toxicity in individuals living with dementia and their care partners.

This study must be considered in the context of its potential strengths and limitations. The strengths of this study center on its novel methodological approach of integrating both qualitative and quantitative methods. In particular, the use of photovoice allows us to richly capture the “patient voice,” which is frequently missing in financial studies that use retrospective, claims-based, and even validated financial questionnaires. Potential limitations include difficulty ascertaining individual insurance plans which impact out of pocket health care expenditures and therefore the degree of financial toxicity. Because recruitment for this study was primarily through partner organizations and social media campaigns, additional limitations may include a bias toward participants of higher socioeconomic positions.

The results of this study have important implications for health care professionals, policymakers, and leaders going forward. Policymakers, including the Center for Medicare and Medicaid Services and Center for Medicare and Medicaid Innovation, have emphasized a focus on addressing affordability of care for people in their strategic vision [38]. However, a major roadblock in developing new care models or policies to improve affordability is robust financial data for people with chronic diseases like dementia. Our study would provide a baseline assessment of the financial well-being of people living with dementia and their care partners to support the development of new strategies for improving costs of care. Furthermore, as new treatments and high-cost drugs are potentially incorporated into the care pathways for those with dementia, this financial baseline will become increasingly important to understand the financial impact these medications and treatments could have. Importantly, understanding financial health at the individual level provides an important foundation for improving health equity. While this work will start with those living with dementia, this protocol could potentially be replicated for people living with other diseases as well and serve as a blueprint for future research efforts in this space. Insights gleaned from this study can also support education and training materials for health care professionals regarding financial resources available for people living with dementia. Gaining a better understanding of the financial challenges that people living with dementia and their caregivers face can inform the creation of pathways to assist families in navigating the financial journey after diagnosis.

### Conclusions

The issue of financial toxicity is an increasingly prevalent problem for people living with chronic conditions and their care partners. Despite considerable evidence for the financial burdens of cancer care, there is little known about financial toxicity’s impact on people living with dementia and their families. This study will use an adapted survey and photovoice interviews as quantitative and qualitative methods to comprehensively characterize financial toxicity in dementia dyads. Study results will enhance the understanding of financial toxicity in individuals living with dementia, provide insights into the primary drivers of financial burden, and explore potential solutions to mitigate this issue. Findings from this study can be used to inform payment reform efforts, lead to improved health outcomes for people living with dementia and increase support for their families.

## Abbreviations

COST: Comprehensive Score for Financial Toxicity
iPCQ: Institute for Medical Technology Assessment Productivity Cost Questionnaire
PIP: pillar integration process
PROMIS: Patient-Reported Outcomes Measurement Information System

## Data Availability

Data sharing is not applicable to this paper, as no data sets were generated or analyzed during the current study.
